# Monitoring of metal content in the tissues of wild boar (*Sus scrofa*) and its food safety aspect

**DOI:** 10.1007/s11356-022-23329-6

**Published:** 2022-09-30

**Authors:** Zoltán Lénárt, András Bartha, Zsolt Abonyi-Tóth, József Lehel

**Affiliations:** 1 Department of Food Hygiene, University of Veterinary Medicine, István u. 2, 1078 Budapest, Hungary; 2 Department of Animal Hygiene, Herd Health and Mobile Clinic, University of Veterinary Medicine, István u. 2, 1078 Budapest, Hungary; 3 Department of Biomathematics and Informatics, University of Veterinary Medicine, István u. 2, 1078 Budapest, Hungary

**Keywords:** Wild boar, Fat, Muscles, Metals, Food safety, Potentially toxic elements

## Abstract

The study was performed on 10 female and 10 male wild boars (*Sus scrofa*) after shooting during the regular hunting season to investigate the concentration of metals in the muscle and fat tissue. The concentrations of essential and non-essential elements were determined (arsenic (As), cadmium (Cd), chromium (Cr), copper (Cu), lead (Pb), manganese (Mn), mercury (Hg), and zinc (Zn)) using inductively coupled plasma optical emission spectrometry. The concentrations of As, Hg, and Cd were below the limit of detection (As, Hg: < 0.5 mg/kg, Cd: < 0.05 mg/kg) in every tissue sample of both sexes. The lead was detected as 0.36 ± 0.16 mg/kg and 0.22 ± 0.06 mg/kg in the muscle of females and males, respectively, showing a significant difference between the sexes (*p* = 0.0184). The measured concentration of Cr was 0.14 ± 0.08 mg/kg and 0.13 ± 0.06 mg/kg, and that of copper was 1.22 ± 0.14 mg/kg and 1.06 ± 0.16 mg/kg in the muscle of females and males, respectively. The same tendency was observed in the case of copper content of fat tissues (female: 0.13 ± 0.10 mg/kg; male: 0.13 ± 0.04 mg/kg; *p* = 0.2707). Manganese concentration of muscle was 0.45 ± 0.30 mg/kg (female) and 1.36 ± 0.96 mg/kg (male), and that of fat tissue was 0.32 ± 0.22 mg/kg (female) and 0.74 ± 0.75 mg/kg (male). The Zn was detected as 56.75 ± 7.86 mg/kg and 1.83 ± 0.76 mg/kg in the muscle and fat of females and 52.12 ± 11.51 mg/kg and 1.94 ± 0.57 mg/kg in males, respectively. Based on data, the consumption of fat and muscle tissues of the wild boars tested can be food toxicologically objectionable, mainly due to the lead content, and thus pose a risk to frequent consumers of this type of game meat.

## Introduction

Sources of environmental pollution are located not only near industrial areas but also in agricultural and natural ecosystems, sometimes away from emission sources (Giżejewska et al. 2015; 2017).

From the point of view of health protection, the polluting effect of metals and their cumulative properties, furthermore, their enrichment in the food chain, are extremely important.

Game meat, as natural food, represents a traditional value in both taste and content (Skibniewski et al. 2015). It is often thought to be “organic” and is often preferred over products produced under intensive conditions.

In Europe, wild boar (*Sus scrofa*), roe deer (*Capreolus capreolus*), and red deer (*Cervus elaphus*) are the most commonly consumed large game species (Falandysz et al. 2005; Ramanzin et al. 2010). At the same time, through pollution, inorganic, organic, and organometallic compounds can affect food quality as harmful factors (Lehel et al. 2016a, b; Taggart et al. 2011). The role of wild animals as an indicator, indicating the extent of environmental pollution, is noted by several authors (Dlugaszek and Kopczyński 2013; Florijancic et al. 2015; Gašparík et al. 2017). Various environmental factors, such as nutrition, condition, age, sex of animals, and sampling (hunting) season, can affect the contaminants’ content of the animal tissues.

The animals can be exposed to toxic levels of different minerals from a wide variety of sources. Industrial and agricultural activities (mining, smelting, etc.) are often associated with local areas of mineral contamination of the water, soil, and air and the plants grown in that area. The accumulation of toxic metals in plants, water, and soil may increase the risk of transfer of them to wild mammals and game animals during nutrition; thus, the high rate of the potentially toxic elements can be taken up by oral route (Falandysz et al. 2005; Lehel et al. 2016a, b; Tyler 2001; Włostowski et al. 2006).

Industry, which was once extremely polluting, continues to be the mainstay of the region’s economy, but has now undergone a significant transformation from a sectoral point of view. It can be said that, in addition to the mechanical engineering sectors, the traditional heavy industrial sectors, such as metal processing, which have higher pollution and load, have remained to this day. The environmental setbacks of the Central Transdanubia Region are unfortunately quite numerous, in addition to the positives. The noise load of many settlements, and with it the air pollution from traffic, is significant, partly due to the lack of roads bypassing the settlements. Furthermore, a significant number of illegal landfills in the region and a significant proportion of landfills do not comply with current legislation. There are a relatively large number of surface and groundwater bases that are sensitive to pollution, and the development of their protection is progressing rather slowly. The environmental awareness of the population of the region is also low compared to other areas.

Using an online distance measurement program, we determined the distance between the sampling sites and the sites of industrial plants in the region to analyze, confirm, or reject potential sources of pollution. According to the calculation, the potential sources of the industrial contaminations are found on average 10–90-km distance.

Wild boars are widely distributed in the world and can be found in Eurasia, the southern part of Asia, and some of the islands of Indonesia. Wild boars, as omnivores, line up their food on a wide scale: mainly plant parts (leaves, roots, seeds, fruits, acorns), invertebrates (insects, earthworms), vertebrates, crops (Lee and Lee 2019; Reginato et al. 2020), and mushrooms (Brzezicha-Cirocka et al. 2016). The concentration of potentially toxic elements and other metals in the tissues of wild boar depends on the dietary intake of these contaminants from different potential sources, especially from their food.

The aim of this study was to determine the content of eight metals (As, Cd, Cr, Cu, Pb, Mn, Hg, Zn) in the muscle and fat tissue of wild boars from the Central Transdanubia Region, Hungary.

## Arsenic (As)

Inorganic arsenic occurs naturally in soil, water, and air and reaches consumers through the food supply due to anthropogenic activities and geological releases. In the past, it was frequently applied in agriculture as herbicides and insecticides and as anabolic for food-producing animals. Arsenic is not an essential part of the human or animal diet. Adverse effects of inorganic arsenic include carcinogenicity, decreased growth, and abnormal reproduction (Lehel et al. 2016a, b; Nigra et al. 2017). In addition, long-term consumption may result in skin lesions, bladder and skin cancer, and neurotoxicity (EFSA 2009, Lehel et al. 2016a, b).

## Cadmium (Cd)

Contamination of soil with Cd can occur from natural sources such as rocks, as well as anthropogenically, with calcareous and phosphorus-containing fertilizers and sewage sludge (Hellström et al. 2007; Bilandžić et al. 2009).

Cadmium is one of the most toxic heavy metals. It is nephrotoxic and carcinogenic and obviously impairs the functioning of several organs (Laczay 2015). Estrogen-like effects may interfere with sexual maturation (Johnson et al. 2003). Cadmium is concentrated in most plants and animals, both in grasses and edible plants, as well as in vertebrates and invertebrates (ATSDR 1999a). Based on structural similarity and physicochemical properties, Cd is able to replace zinc, calcium, and other metals in various metal-containing proteins and enzymes inducing structural and functional changes (Schaefer et al. 2020). Due to its redox activity, it has a detrimental effect on the antioxidant system, causes oxidative stress, increases lipid peroxidation, and changes the lipid composition of the membrane (Xu et al. 2003; Nair et al. 2013; Lehel et al. 2016a, b). The highest amounts of Cd can be measured in the kidneys and liver of food-producing animals (Laczay 2015; Lehel et al. 2017).

Renal toxicity of chronic low-level Cd exposure is pronounced, and tubular necrosis may result in proteinuria, calcuria, aminoaciduria, and glycosuria. Furthermore, Cd uptake can also lead to deregulated blood pressure and other cardiovascular diseases, neurological diseases, and diabetic complications, and it also affects the bone structure, thereby leading to osteoporosis, osteomalacia, and many different organ cancers (Nair et al. 2013).

## Mercury (Hg)

Mercury is ubiquitous in air, soil, and water. A significant portion of atmospheric Hg enters the environment through volcanic activity or industrial activity and is prone to bioaccumulation and biomagnification, thereby posing a threat to food chain safety (Driscoll et al. 2013; Ehrlich and Newman 2008; Sundseth et al. 2017). Mercury in bound form cannot be taken up by living organisms. Some microorganisms can methylate inorganic compounds, resulting in methyl mercury; thus, it can enter the food chain due to its good lipophilic properties (Xu et al. 2019; Nawrocka et al. 2020). Inorganic mercury is nephrotoxic; it may also cause hepatic disorders, various hematological differences, and gastrointestinal alterations. Metallic (elemental) mercury (Hg^0^), inorganic mercury (Hg_2_^2+^ and Hg^2+^), and organic mercury (mostly methyl mercury) are the chemical forms of mercury (JECFA-959 2011).

## Lead (Pb)

The effects of lead on various organ systems are a widely investigated area. Exposure to lead contamination can be manifested in lead-induced encephalopathy (Eastman and Tortora 2020), gastroenteritis, and peripheral nerve degeneration (Bilandžić et al. 2009). Lead can decrease the production of erythrocytes and increase the destruction of erythrocytes, resulting in anemia, and Pb has harmful effects in renal and gastrointestinal perspectives as well (Halmo and Nappe 2020).

Furthermore, lead can modify the spermatozoa quality (count, motility, viability, altered morphology); thereby, it can reduce the fertility of males in experimental animals (rats, mice, avians, and rabbits). In females, Pb can cause deviations such as prolonged or/and irregular cycle, decreased follicular growth, increased number of atretic follicles, and degeneration of the corpus luteum (Massányi et al. 2020).

## Chromium (Cr)

Recent studies suggest that chromium is no longer among the essential metals. Based on the investigation of Di Bona et al. (2011), a diet with as little chromium as possible had no effect on glucose metabolism, insulin sensitivity, or body weight compared to the other experimental groups where a certain amount of chromium was present in the diet in rats.

Hexavalent chromium (Cr(VI)) compounds are mutagenic and carcinogenic (Vincent and Lukaski 2018); furthermore, the main toxic effects were small intestinal damage and anemia in experimental animals (FSCJ 2019). In contrast, trivalent chromium (Cr(III)) was considered by different scientists to be an essential element in mammals. It has been postulated to be involved in regulating the metabolism of carbohydrates and lipids by increasing the effectiveness of insulin (Vincent and Lukaski 2018).

Meat and meat products, oils, and fats are rich in Cr, but most foods contain this metal. Median dietary chromium intakes were estimated at 57.3–83.8 µg/day in adults (≥ 18 years) using food consumption and body weight data during a large-scale survey (EFSA 2014).

## Copper (Cu), manganese (Mn), and zinc (Zn)

The specified essential trace elements (Cu, Mn, Zn) are performing different biochemical functions and are involved in cell metabolism and regulation. All three elements are acting as antioxidants and as enzyme cofactors. Manganese and copper are participating in redox reactions, and zinc is participating in cell signaling processes (Carpenè et al. 2020). These three metals are human nutritional requirements, so the food consumed must contain enough quantity of them.

## Material and methods

### Location of the sampling

**Fig. 1:**
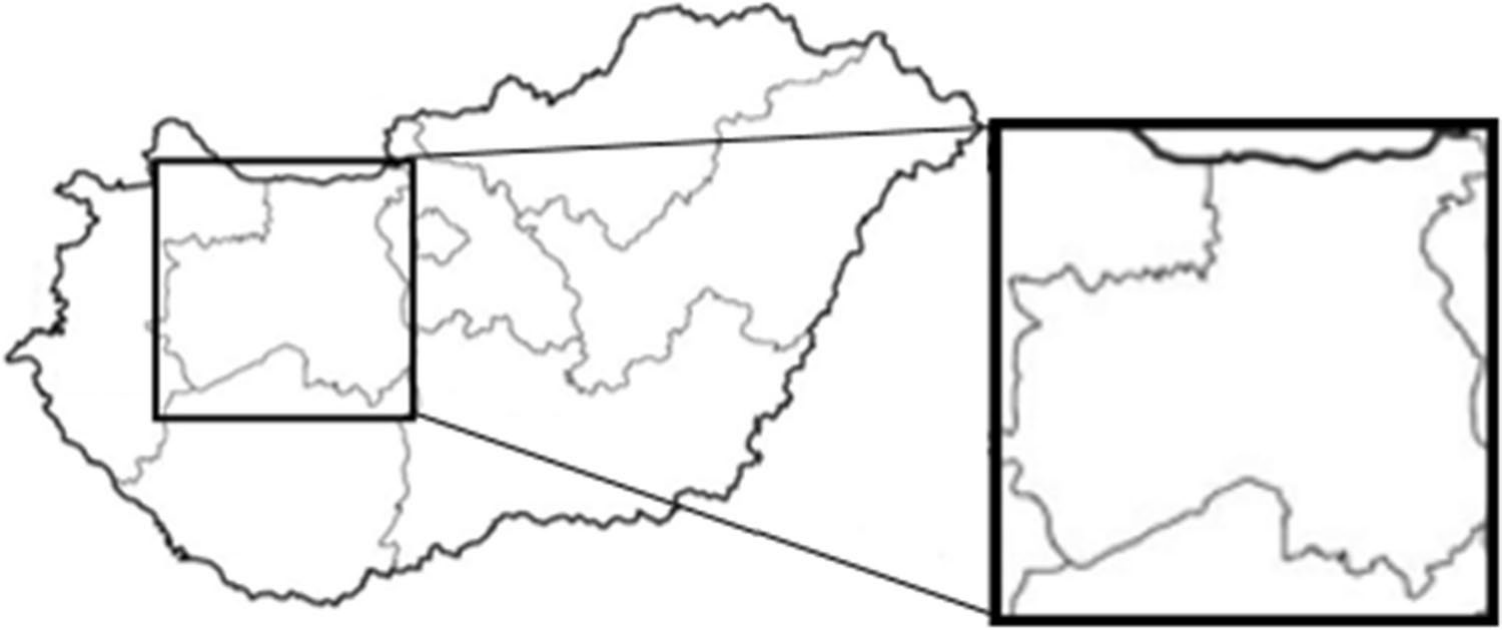
Origin of the samples: Central Transdanubia Region including Komárom-Esztergom, Fejér, and Veszprém county

The study was performed on 20 wild boars including of both sexes (10 males, 10 females) living in a landscape with forest and land used for agricultural purposes in different hunting areas within a radius of 100 km in Hungary, Central Transdanubia Region (Fig. 1) including Fejér county, Komárom-Esztergom county, and Veszprém county.

The regular hunting season for wild boars is in a whole year based on legal regulation (National Regulation 2004). Sampling was performed in 2018 August and September.

The mean age of all investigated animals was 3.5 years (4.1 years for males, 2.8 years for females). The age of the animals was determined based on the assessing the degree of tooth eruption and wear and tear of teeth of the lower jaw, the body weight of the animals, and their color/color changes (Magnell and Carter 2007; Merta et al. 2015). The youngest individual was approximately 1 year old, and the oldest was 6 years old.

### Sampling

The samplings were performed on the spots during the official hunting season. Thus, permission is not necessary based on the legal regulation of animal welfare protection.

A muscle sample of 2 g was taken from the gluteal part (*musculus biceps femoris*) of each wild boar (instead of the cervical part which may be contaminated due to the bullet during the shooting) using a ceramic knife to avoid the possibility of possible contamination with a metal device. The sampling procedure of the fat tissue from the *capsula adiposa renis* was the same.

Each sample was put in a well-sealed, pre-labeled plastic bag, and both tissues of the animals were placed in a large bag. All samples were placed in a portable cooler after sampling until transportation of them to the analytical laboratory and where they were stored at − 18 ℃ in a freezer until analysis.

### Analytical method

#### Chemicals used and preparation of sample

Muscle and fat tissue samples were digested with a mixture of analytically pure concentrated nitric acid (69 w/w%, Aristar) and hydrogen peroxide (30 w/w%, Normapur) using a microwave digester (CEM MARS6, CEM Corporation, USA). Prior to the measurements, the laboratory glass and plastic devices were cleaned with 0.15 M hydrochloric acid (37 w/w%, Aristar) and then rinsed with high purity deionized water prepared using a Purite Select Fusion 160 BP water purification system (Purite Ltd., England), so that contamination with laboratory equipment was avoided.

Half (0.5) g of each tissue sample was weighed into CEM MARS6 Express Teflon dishes. Subsequently, 5 ml of nitric acid and 5 ml of hydrogen peroxide were added, and the exploration program was started. The excavation parameters were as follows: ramp: 35 min; temperature: 200 ℃; hold: 50 min; energy: 1700 W. In the next step, the samples were made up to 25 ml with ultrapure water, and after heavy dilution, the potentially toxic elements were measured. A 1 mg/l Y solution (VWR International Ltd., England, Leicestershire) was used as an internal standard for the measurement, and a 0.25 mg/l gold solution (VWR International Ltd., England, Leicestershire) was used to stabilize the mercury content. Blank and QC (quality control) samples were prepared by the same method.

Calibration of ICP-OES was performed with ICP multi- (Perkin Elmer Inc., USA, Shelton) and mono-element (VWR International Ltd., England, Leicestershire) standards. Argon gas of 4.6 purity (Messer Hungarogáz Kft., Hungary) was used for the measurements. Bovine liver (NIST-1577C) standard reference material (NIST, USA) was used to control the measurements (QC samples).

#### Analytical measurements

The concentration of the potentially toxic elements (As, Cd, Hg, Pb, Cr, Cu, Mn, Zn) was determined using a Perkin Elmer Optima 8300 DV type (Perkin Elmer, USA) inductively coupled plasma optical emission spectrometer (ICP-OES).

Calibration curves were recorded by the analytical equipment in the range of 0 to 200 mg/kg. Limit of detection of the tested metals was 0.2 mg/kg for lead, 0.5 mg/kg for arsenic and mercury, and 0.05 mg/kg for cadmium (0.05 mg/kg), chromium, copper, manganese, and zinc. When determining the battery contents, internal quality control was ensured by measuring known metal-containing QC samples at least 10 times. Leaving the extreme values, the standard deviation was determined, which had to remain within ± 15% of the nominal concentration value for the QC measurements to be valid. The authentic Hg content of the QC standards was less (0.00536 mg/kg) than the limit of detection. The Hg content of the test samples was also below the detection limit.

### Statistical analysis

The data of the obtained measurement results were processed using Microsoft Excel and the R-program (version 3.1.3). The mean and standard deviation of the potentially toxic elements in both muscle and fat tissue were calculated for both sexes. Values below the LOD were replaced by the detection limit during the calculations (except for the lead level measured in male fat). The concentrations of As, Cd, and Hg were below the LOD in all samples of both sexes; therefore, they were not analyzed statistically.

Where possible, a two-sample *t*-test was used. Where there were too many values below the detection limit for the mean estimate to be good enough, the Wilcoxon rank-sum test for medians was used.

Heavy metal contents in the fat and muscle were compared by paired *t*-test because both values were measured in all animals. Here, the effect of sex was not considered.

For the statistical evaluation of the lead content of fat samples, Fisher’s exact test was used to compare the concentrations between the sexes because only one sample was above the detection limit.

For calculation of the possible metal loading or uptake by human consumers, our results of metal concentrations were compared with the recommended daily or weekly intake (PTDI/WI: provisional tolerable daily/weekly intake).

The weekly intake was estimated based on the lead concentration measured in our muscle and fat samples and an average meat consumption value (200 g). The detected concentrations, expressed in mg/kg, were divided by the mean meat consumption value, and the result was further divided by the body weight of an average adult (60 kg) and multiplied by 7 (days/week) (EMEA 2001). For chromium, copper, manganese, and zinc, only the daily value had to be calculated. All this could be counted for Pb, Cr, Cu, Mn, and Zn, as the values for As, Cd, and Hg were below the detection limit.

## Result and discussion

The detected concentrations of the potentially toxic elements (As, Cd, Cr, Cu, Hg, Mn, Pb, Zn) in the muscle and fat tissues of wild boars are presented in Table 1.

**Table 1:** Concentration of metals in the muscle and fat tissue of wild boars (mean ± SD, mg/kg w.w.)

Tissue	Sex	Number of samples	As	Hg	Cd	Pb	Cr	Cu	Mn	Zn
Muscle	Female	10	< 0.5	< 0.5	< 0.05	0.36 ± 0.16	0.14 ± 0.08	1.22 ± 0.14	0.45 ± 0.30	56.75 ± 7.86
	Male	10	< 0.5	< 0.5	< 0.05	0.22 ± 0.06	0.13 ± 0.06	1.06 ± 0.16	1.36 ± 0.96	52.12 ± 11.51
	Statistical difference		-	-	-	0.0184^a^	0.7526^b^	0.0739^b^	0.0159^b^	0.3092^b^
Fat	Female	10	< 0.5	< 0.5	< 0.05	< 0.2	0.08 ± 0.07	0.13 ± 0.10	0.32 ± 0.22	1.83 ± 0.76
	Male	10	< 0.5	< 0.5	< 0.05	< 0.2	0.08 ± 0.07	0.13 ± 0.04	0.74 ± 0.75	1.94 ± 0.57
	Statistical difference		-	-	-	1^c^	0.8645^a^	0.2707^a^	0.1155^b^	0.7198^b^

^a^Wilcoxon test

^b^Two-sample *t*-test

^c^Fisher’ exact test

### Muscle tissue

The concentration of arsenic was below the limit of detection (LOD) (< 0.5 mg/kg) in every tissue sample of both sexes.

Basically, the maximum permissible level of arsenic is not regulated for mammal food-producing animals in the EU legal order for foodstuffs of animal origin, including muscle and fat tissue (Regulation (EC) No 1881/2006). In Slovakia, 0.31 mg/kg arsenic concentration was detected in the muscle of wild boar (Piskorová et al. 2003). Similarly to our study, low arsenic concentrations (0.013–0.019 mg/kg) in wild boar tissues were found in Croatia (Florijancic et al. 2015). Based on the detected concentrations of As, it is favorable from a food toxicological point of view because a small amount of arsenic enters the food chain in the examined area.

The concentration of mercury was also below the limit of detection (LOD) (< 0.5 mg/kg) in every tissue sample of both sexes. Contrary to our results in a Polish study, Hg was detected in several tissue samples of wild boars living in three different areas. The detected values were 0.007 ± 0.006 mg/kg, 0.007 ± 0.005 mg/kg, and 0.006 ± 0.004 mg/kg (Durkalec et al 2015). Similarly to arsenic, the maximum level of Hg is not specified in the Commission Regulation (EC) no1881/2006 (EC 2006). Based on a suggestion made by The European Union Reference Laboratory for Chemical Elements in Food of Animal Origin in Rome (EU-RL CEFAO), the maximum level of Hg in the Commission Regulation (EC) no 149/2008 should be respected (EC 2008). In this regulation, the maximum level of Hg (0.01 mg/kg of wet weight [w.w.]) is specified for residues of pesticides in or on food and feed of plant and animal origin (Durkalec et al. 2015). A comprehensive study also measured mercury concentrations in wild boar muscle tissue; 600 animals were tested, of which 40 samples contain mercury were below the limits of quantification. The mean value was 0.0056 ± 10.2 mg/kg w.w. (Nawrocka et al. 2020).

The concentration of cadmium was below the LOD (< 0.05 mg/kg) in our study. There is no maximum permissible EU-regulated level of Cd in game meat, although in livestock meat products, the maximum level for cadmium is 0.05 mg/kg (EC 2006). The detected cadmium values were in the range of 0.009–0.02 mg/kg in the muscle of wild boars in Romania, Harghita county (Raicu et al. 2020), which shows similar concentrations to our study. In another study, an average of 0.0078 mg/kg of cadmium was obtained in wild boar muscle, which may be like ours at a lower detection limit (Danieli et al. 2012). Values below the LOD findings were noted in the muscle samples of roe deer (*Capreolus capreolus*) (Lehel et al. 2016a, b), not much differently in the muscle of roe bucks 0.04 ± 0.02 mg/kg (Lehel et al. 2017). However, higher residue was detected in liver and kidney in both bucks (0.13 ± 0.04 mg/kg; 1.03 ± 0.52 mg/kg) and does (0.03 ± 0.02 mg/kg; 0.21 ± 0.20 mg/kg) (Lehel et al. 2017).

In our investigation, the concentration of lead in the muscle of both sexes exceeded the maximum limit (0.1 mg/kg). However, there was a significant difference between the sexes (*p* = 0.0184). Lead was detected in 70% of sow (female) muscle samples, and the mean value (0.36 ± 0.16 mg/kg) and the individual sample concentrations (0.20–0.67 mg/kg) exceeded the official limit value (0.1 mg/kg). In males, on the other hand, lead was detected in 20% of the samples above the LOD.

The concentrations of the potentially toxic elements in different tissues of the animals can be influenced by several factors, such as the age and gender of the animals, the eating habit of the animals, physical–chemical properties of the metals, and their distribution in natural environmental sources and industrial and agricultural activities at the area (ATSDR 1999b, Combs 1987, IPCS 1989, 1995, Wren 1986). In our study, the average age of the females was 2.8 years compared to males (4.1 years). Young/younger animals can show a higher concentration of Pb in the hair than adults without statistical significance (Oropesa et al. 2022) due to their habit of living with sows, and the youngers have to exploit the rest of plants (Clutton-Brock and Albon 1989).

Similarly, the higher concentrations of Pb in the hair of younger animals in comparison with adult boars can be explained by eating habits (Tack 2018).

Furthermore, the difference may be due to metabolic or physiological aspects between the two genders explained by different distribution of metals. The metals can be distributed to the fetuses during pregnancy or to progeny during lactation (Oskarsson et al. 1992, Pérez-Carrera et al. 2016). However, even the accumulated metals (e.g., Pb) can be mobilized from the storage site (e.g., bones) during pregnancy and lactation (Lehel et al. 2016a, b).

Tissue contamination may have been caused by environmental contamination due to industrial production in the area. Higher lead concentrations were found in samples collected around settlements that were, on average, 38.2 km and 51.2 km as the crow flies from industrial facilities.

A significant difference was found in the lead concentration of muscle samples between males and females. In contrast, there was no statistically significant difference in western Slovakia when comparing the two sexes. The median concentration of lead was found at 0.44 mg/kg (min.: 0.04 mg/kg, max.: 61.30 mg/kg) in the muscle tissue of wild boars (Gašparík et al. 2017). However, it was higher than our values. Similar to our results, the average measured lead concentration was 0.29 mg/kg (extreme: 0.05–0.77 mg/kg) in wild boar meat in Poland, indicating that a significant proportion of the samples were unfit for consumption according to the applicable limits (Dlugaszek and Kopczyński 2013). Lower concentrations of Pb were found in Italy in the meat of wild boar (0.13 ± 0.03 mg/kg), exceedingly slightly below the current limit (Amici et al. 2012). Similarly, Danieli et al. (2012) measured 0.12 ± 0.03 mg/kg of lead in wild boar muscle in Italy.

In recent years, lead levels had been measured in huntable ruminants in Hungary. A study reported high lead concentrations that were found in the muscle of roe deer. The median value of Pb was 0.39 mg/kg in roebuck and 1.32 mg/kg in doe, where 60% (roebuck) and 90% of the samples (doe) were above the limit (Lehel et al. 2017). The concentration of lead was found to be 0.48 ± 0.21 mg/kg in another Hungarian study, as well as in the muscle of roe deer (Lehel et al. 2016a, b).

The chromium concentration was 0.14 ± 0.08 mg/kg and 0.13 ± 0.06 mg/kg in the muscle of females and males, respectively. Basically, today, there is no defined population reference intake and average requirement for chromium (EFSA 2014).

An average chromium level of 0.133 mg/kg was measured in the same species, which is consistent with our results (Danieli et al. 2012).

The amount of copper ranged from 0.73 to 1.47 mg/kg in muscle tissue of wild boars in our study. The detected concentration of Cu in the muscle of females was 1.22 ± 0.14 mg/kg and 1.06 ± 0.16 mg/kg, below the regulated limit value.

In Slovakia, 1.62 mg/kg Cu (0.94–2.51 mg/kg) was noted in the same species which is more than what we measured (Gašparík et al. 2017). In central Poland, Dlugaszek and Kopczyński (2013) measured similar values as we did, and a mean copper concentration was 0.92 mg/kg (0.15–1.79 mg/kg) in wild boar muscle. Higher values were detected in Italy; 12.20 ± 4.73 mg/kg of copper was noted in the muscles of wild boars (Amici et al. 2012). Examining the content values of wild boar meat, copper was measured at a concentration of 0.4 mg/kg in Latvia (Strazdina et al., 2014).

Higher concentrations were noted in ruminants such as in European deer muscle tissue (*Capreolus capreolus*) at an average of 1.65 mg/kg (extreme: 0.43–5.36 mg/kg) and in red deer (*Cervus elephus*) meat (average: 11 mg/kg; extreme value: 5.7–22 mg/kg) (Dlugaszek and Kopczyński 2013; Jarzyńska and Falandysz 2011). Giżejewska et al. (2017) determined the concentration of Cu in tissues of free-ranging red deer in Poland and find an average of 7.29 ± 7.02 mg/kg Cu in the liver, 4.08 ± 0.45 mg/kg Cu in the kidney, and 1.10 ± 0.35 mg/kg Cu in the muscle tissue.

In the liver of wild boar, a higher concentration of Cu was measured in females (8.54 ± 2.64 mg/kg) and males (7.75 ± 2.12 mg/kg) (Kasprzyk et al. 2020).

In our study, Mn concentration in the muscle of wild boar was 0.45 ± 0.30 mg/kg in females and 1.36 ± 0.96 mg/kg in males, respectively. A study examined a comparison analysis of the main chemical composition parameters of wild boar meat and pork (Skobrák et al. 2011). It was found that wild boar meat contained 0.22 ± 0.04 mg/kg Mn in extensively housed individuals which is slightly lower than our result (Skobrák et al. 2011). This study also examined concentration of Mn in wild boars kept in semi-intensive (0.57 ± 0.12 mg/kg) and intensive (0.52 ± 0.052 mg/kg) conditions. The soil of the extensive park contained lower manganese than the semi-intensive and intensive parks (143 mg/kg vs. 314 mg/kg and 484.5 mg/kg) (Skobrák et al. 2011). The level of Mn in the wild boar groups did not differ significantly and corresponded to the values published about pork: 0.504 ± 0.01 mg/kg (Guang-Zhi et al. 2008), 0.43 mg/kg (Gerber et al. 2008), and 0.12 ± 0.06 mg/kg (Leibholz et al. 1962). In the liver of free-living wild boars, a higher concentration of Mn was measured at 2.85 ± 0.72 mg/kg in females and 2.83 ± 0.44 mg/kg in males (Kasprzyk et al. 2020).

The concentration of zinc was 56.75 ± 7.86 mg/kg in the muscle samples of females and 52.12 ± 11.51 mg/kg in males. In extensive conditions, the concentration of zinc was 52.17 mg/kg in wild boars, nearly the same as our value (Skobrák et al. 2011). Among the analyzed microelements, the presence of zinc 16.13 mg/kg in the muscle tissue, respectively, was detected in domestic pigs (Stasiak et al. 2017). The concentration of Zn in the liver of wild boar was closely similar in females (54.73 ± 14.39 mg/kg) and males (62.77 ± 12.00 mg/kg) (Kasprzyk et al. 2020). The detected concentration of Zn was 67.14 ± 15.67 mg/kg in the muscle of red deer in Poland, which is slightly higher than that detected by us in wild boars (Giżejewska et al. 2017).

### Calculated metal intakes

In the case of lead, the PTWI value established by the FAO/WHO is 25 μg/kg (JECFA-960, 2011; Laczay, 2015). According to our calculations (Table 2), a 60-kg adult man consumes an average of 6.85 μg/kg of lead, which is 27.4% of the PTWI value, in the case of muscle tissue, with an average game consumption of 200 g. This is safe for weekly consumption. There was no significant difference between the sexes; the mean value was 8.51 µg/kg in sows and 5.17 µg/kg in males. Thus, if only the meat of one or the other is consumed, PTWI would account for only 34.0% (female) and 20.7% (male), respectively. In 11 samples, the lead level was below the detection limit, so we calculated a value of 0.2 in these cases. The PTWI value for the sample with the highest lead concentration (0.668 mg/kg Pb) is only 62.3% of the FAO/WHO value. For fat tissue, PTWI calculation could not be performed.

**Table 2:** Calculation of weekly (Pb) and daily (Cr, Cu, Mn, Zn) intake of metals based on the detected concentrations (mg/kg)

	Intake of metals (μg/kg)				
	Weekly	Daily			
	Pb	Cr	Cu	Mn	Zn
Muscle					
Gender					
Female	8.51 ± 3.76	0.44 ± 0.20	4.05 ± 0.55	1.51 ± 0.99	189.11 ± 26.19
Male	5.17 ± 1.17	0.48 ± 0.27	3.53 ± 0.68	4.54 ± 3.19	173.67 ± 38.37
All	6.85 ± 3.21	0.46 ± 0.24	3.79 ± 0.62	3.02 ± 2.09	181.39 ± 32.28
Fat					
Gender					
Female	4.76 ± 0.00	0.27 ± 0.22	0.45 ± 0.43	1.05 ± 0.73	6.10 ± 2.54
Male	9.64 ± 15.72	0.26 ± 0.24	0.44 ± 0.19	2.46 ± 2.50	6.47 ± 1.90
All	7.16 ± 11.11	0.26 ± 0.23	0.45 ± 0.31	1.76 ± 1.61	6.28 ± 2.22

Tolerable upper intake level of chromium is not determinable owing to a lack of data on a specific toxicological adverse effect (Oria et al. 2019).

For the muscle samples, the calculated metal uptake (0.46 ± 0.24 μg/kg) covers only 2.3% of our chromium requirement, and the chromium content of fat (0.26 ± 0.23 μg/kg) is only 1.3%. Calculated with a daily intake of 20 µg of chromium (EFSA 2015). In this case, there was virtually no difference between the two sexes.

For arsenic, cadmium, and mercury, the calculation could not be performed because we had all values below the detection limit. PTWI value of inorganic arsenic is not regulated in the EU; the previous PTWI value has been withdrawn (15 µg/kg body weight [bw]) (JECFA-959 2011). The cadmium content in pork is also regulated by Regulation 1881/2006/EC, where the maximum permissible value is 0.05 mg/kg. PTWI for cadmium was stated as 7 μg/bw (JECFA 983 2013). For Cd food intake, there was specified a tolerable weekly intake (TWI) as 2.5 μg/kg bw by the EFSA CONTAM Panel (EFSA 2009; Ciobanu et al. 2020). Mercury is also not regulated in this regard; 1.6 μg/bw PTWI for methylmercury had been published (JECFA-959 2011).

Our concentrations of copper, manganese, and zinc are quite low compared to the recommended daily intakes, and for an adult human body, we can say that wild boar meat and fat are not considered significant sources of intake.

The recommended daily intake for copper is 1.6 mg (1600 µg) for men and 1.3 mg (1300 µg) for women (EFSA 2015). The Hungarian regulations are based on 49/2014. (IV. 29.) VM, regulating the amount of copper and providing for an upper limit for game meat and food products made from it, in the amount of 5 mg/kg (49/2014. (IV. 29.) VM).

Based on a 60-kg adult consumer and 200 g of consumed tissue, 3.79 μg/kg of copper enters the body and there was no significance to the sex of the animals (4.05 μg/kg (female) and 3.53 μg/kg (male). Based on the calculated metal uptake, the amount of metal introduced covers a very small part of the daily copper need: In the case of wild boar meat, 0.24% of the needs of men and 0.29% of the needs of women; for wild boar fat, 0.03% of men’s needs and 0.04% of women’s needs.

The National Institutes of Health recommends a daily intake of 2.3 mg (2300 µg) of manganese for men and 1.8 mg (1800 µg) for women. The adequate daily zinc intake is 10.1 mg (10,100 µg) for men and 8.2 mg (8200 µg) for women (NIH 2010). The manganese consumption calculated from the metal content of our muscle samples covers 0.13% of the demand of men and 0.17% of the demand of women. Fat samples provide 0.08% of demand for men and 0.10% for women.

PMTDI (provisional maximum tolerable daily intake) 0.3–1 mg/kg/bw/day for zinc (JECFA-683 1982). The zinc consumption calculated from the metal content of our muscle samples covers 1.80% of the demand of men and 2.21% of the demand of women. Fat samples provide 0.06% of demand for men and 0.08% for women.

### Fat tissue

While the maximum limits of Cd and Hg in fat tissue are not regulated, the value of it is 0.1 mg/kg for Pb in the fat of animal origin under regulation 1881/2006/EC.

In our study, the concentration of arsenic, cadmium, and mercury was below the limit of detection (LOD) (As, Hg: < 0.5 mg/kg; Cd: < 0.05 mg/kg) in every fat sample of both sexes. We obtained values below the LOD, so we could not apply statistical tests. Due to the concentrations below detection limits, the lead content of muscle and fat samples was not comparable with any of the statistical tests.

Based on the investigation of Mazzocco et al. (2020), mice fed with a high-fat diet compared to a low-fat diet group showed significant increases in Cd levels.

In another survey conducted in Hungary, Cd was detected in very low concentrations in the fat of roe deer, and very high Pb concentrations were detected in three samples assuming contamination with projectiles. The detected Pb concentrations in fat samples were 2.93 ± 6.26 mg/kg in roebuck and 11.57 ± 27.30 mg/kg in doe (Lehel et al. 2017).

Zhang et al. (2019) published that As exposure causes gender-dependent changes in high-fat diet-induced kidney damage.

In mice, a high-fat diet increased arsenic-induced heart injury (Ahangarpour et al. 2018) and arsenic-induced lung damage through oxidative stress (Hemmati et al. 2018). Markowski et al. (2011), as part of their complex study, stated that arsenic does not appear to be retained in fat tissue after exposure is terminated. That study examined tissue and dose-specific arsenic accumulation in mouse offspring following maternal consumption of As.

Kawakami et al. (2010) studied the concentrations of mercury in three kinds of tuna (wild bluefin tuna, farmed bluefin tuna, and farmed southern bluefin tuna) with various fat contents (akami, chutoro, otoro). The concentrations of mercury in the leaner part of the tuna (akami) were 0.42 µg/g and 0.36 µg/g in the “intermediate” part of the tuna (chutoro) and 0.31 µg/g in otoro with the highest fat content. It is clear that the mercury content was inversely proportional to the fat content of the tuna.

High-fat diet significantly affected the levels of some essential metals, including chromium, copper, and zinc, in mice brains (Mazzocco et al. 2020).

In this study, the concentration of copper in fat tissue was 0.13 ± 0.10 mg/kg in female and 0.13 ± 0.04 mg/kg in male (*p* = 0.2707). The concentration of Mn in the fat tissue was 0.32 ± 0.22 mg/kg in females and 0.74 ± 0.75 mg/kg in males. Zinc content of the fat was 1.83 ± 0.76 mg/kg (female) and 1.94 ± 0.57 mg/kg (male).

## Conclusions

It would continue to be important to reduce pollution, possibly rethinking and revising the regulation of potentially toxic elements and other environmental contaminants on the food of animal origin. From an environmental approach, the wild boar reside can be contaminated, and this could lead to the accumulation of certain environmental pollutants in their tissue.

The maximum permissible amount of lead in meat and fat is laid down in Regulation (EC) No 1881/2006 as 0.1 mg/kg. PTWI, in this case, is set at 25 μg/bw (JECFA-960 2011).

The potential health hazards can be indicated with these calculated PTWI values (JECFA-776 1989; JECFA-959 2011; JECFA-960 2011).

Generally, it can be stated that the food safety risks arising from the consumption of wild boar tissues cannot be considered reassuring or negligible due to the exceeding of the maximum permissible concentrations based on our study.

The consumption of tissues, especially muscle of the wild boar investigated, is objectionable from a food safety aspect based on our data, and it poses a risk to the consumer due to the lead contents over the legal tolerable limits.

The measured residue concentration of As, Cd, and Hg was below the LOD in the investigated tissues of wild boar, indicating no health risk for the consumers. The residue of lead in the muscle (0.36 ± 0.16 mg/kg in females, 0.22 ± 0.06 mg/kg in males) exceeded the maximum limit established for domesticated pigs, but its calculated weekly intake was below the PTWI.

## Data Availability

The datasets used and/or analyzed during the current study are available from the corresponding author upon reasonable request.
